# Assessing Patient Satisfaction by Measuring Service Quality in Outpatient Service of a Public Hospital in Bagmati Province, Nepal

**DOI:** 10.1002/puh2.70143

**Published:** 2025-10-27

**Authors:** Vivek Uprety, Binita Kumari Paudel, Mallika Shrestha, Sagun Kharel, Sunil Neupane, Hemanta Neupane, Manoj Sigdel, Bipindra Pandey

**Affiliations:** ^1^ Department of Public Health, Purbanchal University School of Health Sciences Purbanchal University Morang Nepal; ^2^ Department of Nursing Madan Bhandari Academy of Health Sciences Hetauda Nepal; ^3^ Nobel College Pokhara University Kathmandu Nepal; ^4^ IIHMR University Jaipur Rajasthan India; ^5^ Department of Pharmacy Madan Bhandari Academy of Health Sciences Hetauda Nepal

**Keywords:** expectation, Nepal, outpatient department, patient satisfaction, perception, public hospitals, service quality (SERVQUAL) model

## Abstract

**Introduction:**

Patient satisfaction, a key healthcare objective, depends on various factors, including service quality (SERVQUAL), medication availability, staff behavior, cost, infrastructure, comfort, emotional support, and respect for preferences. Dissatisfaction arises when healthcare services fail to meet patients’ expectations and perceptions. Therefore, this study aimed to determine patient satisfaction using a satisfaction‐by‐gap model.

**Materials and Methods:**

This hospital‐based cross‐sectional study randomly selected 311 patients visiting the outpatient department (OPD) using systematic sampling from a public hospital. Data were collected via interviewer‐administered surveys using the SERVQUAL model. Correlation and multiple linear regression analyses conducted using IBM SPSS 16.0 revealed significant associations at *p* < 0.05.

**Results:**

The results indicated that 70.1% of the patients were female and 23.8% were in the 40–49 years age group. Moreover, 52.7% had visited the OPD four or more than four times. The results revealed gaps between patients’ expectations and perceptions in all five dimensions. The findings suggest that among the five dimensions, tangible factors are the most important predictors of patient satisfaction. Patients are more likely to report higher levels of satisfaction when they perceive high levels of empathy, responsiveness, assurance, and reliability in healthcare services.

**Conclusion:**

This study assessed patient satisfaction by evaluating SERVQUAL in a public hospital in Bagmati Province, Nepal. Patients’ perceptions of hospital services were significantly linked to overall satisfaction, with reliability, responsiveness, assurance, empathy, and tangible aspects of care being key predictors. Enhancing these areas can positively impact patient satisfaction and healthcare outcomes.

## Graphical abstract

This study assessed service quality in a public hospital in Bagmati Province, Nepal, using the SERVQUAL model. Tangible factors such as facilities, equipment, and staff appearance emerged as the strongest predictors of patient satisfaction, underscoring the importance of improving hospital infrastructure to enhance patient care.

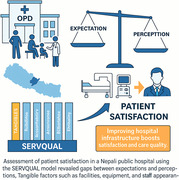

**Abbreviations**
OPD: outpatient department
SERVQUAL: service quality

## 1 Introduction

Good health is both a basic human need and an essential right. The responsibility of providing fair and comprehensive healthcare to all citizens lies with the government and medical professionals. However, current healthcare services often fail to meet patient expectations and requirements. In recent times, international organizations such as the World Bank have advised less‐developed nations to focus on maximizing the impact of their limited resources on public health. This approach aims to achieve optimal health outcomes at affordable costs while ensuring that healthcare services are responsive to the preferences and demands of patients [1]. A hospital is an established, staffed, and equipped facility to diagnose illnesses, provide medical and surgical treatment for the sick and injured, and accommodate patients throughout their care. Modern hospitals often serve as centers for research and education as well [2]. Both nursing and medical professionals prioritize patient care. Although technological advancements have influenced the dimensions of patient care over time, their fundamental aspect remains unchanged: delivering exceptional care to patients [3]. Patient satisfaction is a crucial objective of any healthcare system and is influenced by various factors. These include the quality of clinical services, medicine availability, healthcare providers’ behavior, service costs, hospital infrastructure, physical comfort, emotional support, and respect for patient preferences [4, 5]. Evaluating patient satisfaction helps to identify and address gaps in providing healthcare services that are effective, efficient, accessible, acceptable, equitable, and safe [6]. Patient satisfaction is defined as the pleasure or contentment that patients experience when using a health service. The most significant factors for delivering high‐quality services are customer expectations and requirements [7]. These expectations stem from the ideal standards imagined by patients or their previous experiences with services. When actual perceived services align with these expectations, patients become satisfied with the care provided [8]. The main objective of this study was to assess patient satisfaction by measuring the perceived service quality (SERVQUAL) gap among patients receiving outpatient department (OPD) services in public hospitals.

## 2 Materials and Methods

A cross‐sectional study conducted in a public hospital in Bagmati Province, Nepal, utilized the SERVQUAL model as a quantitative instrument to evaluate patient satisfaction. The study included individuals aged ≥18 years who used any OPD service, excluding pediatric care. A previous study at Chitwan Medical College's OPD revealed an overall satisfaction rate of 75.9%, with a mean score of 24.19 ± 2.92 [9]. Consequently, the proportion of outpatient satisfaction (*p*) was set at 0.759, and the maximum allowable error was established at 0.05, with a 95% confidence interval, resulting in a *z* value of 1.96. *q* = (1 − *p*) or proportion of all unsatisfied OPD patients, which is 1 − 0.759 = 0.241. The sample size of 311 was determined using the formula *z* ^2^ *pq*/*d* ^2^, where *p* = 0.759, accounting for a 10% nonresponse rate. Systematic random sampling was employed to recruit patients, selecting every fifth unit in the sampling frame (first, sixth, 11th, 16th, 21st, and so on). This study included all patients who met the inclusion criteria and provided informed consent. A validated and standardized SERVQUAL model was utilized to assess patient satisfaction, identify five SERVQUAL dimensions, and employ a five‐point Likert scale [10]. The mean gap scores were calculated for each SERVQUAL domain and patient satisfaction. The SERVQUAL model was chosen for this research because it is one of the most extensively validated and utilized tools for evaluating SERVQUAL in the healthcare sector. Unlike other models that emphasize structural or outcome‐based metrics, SERVQUAL assesses the discrepancy between patient expectations and their perceptions across five essential dimensions: tangibility, reliability, responsiveness, assurance, and empathy [12]. This makes it especially effective for capturing patients’ subjective experiences, which is crucial for assessing their satisfaction in outpatient environments. Furthermore, SERVQUAL has been widely applied in both developed and developing countries, enabling the comparability of results and enhancing the external validity of the findings [7, 15]. The capacity of the model to quantify service gaps offers actionable insights for hospital administrators to identify key areas for improvement. For these reasons, SERVQUAL was favored over other models such as Donabedian's structure–process–outcome framework or patient‐reported outcome measures, which, while valuable, may not capture service delivery perceptions with the same level of detail and patient‐centered focus [18].

The study ensured voluntary participation and obtained informed consent from all participants, with approval from the Institutional Review Committee of Purbanchal University. Data analysis was conducted using SPSS (Windows version 16.0), and correlation analysis was applied to examine the relationships between the variables and satisfaction. Multiple linear regression analysis was used to investigate the connection between multiple independent variables (demographic factors and five service dimensions) and the dependent variable (patient satisfaction), as well as to predict the outcomes of the independent variables. Statistical significance was set at *p* < 0.05.

## 3 Results

This study comprises multiple sections that offer crucial insights into how SERVQUAL factors correlate with patient satisfaction. This investigation examines the connection between SERVQUAL elements, which are based on independent variables such as socio‐demographic characteristics and SERVQUAL dimensions, and the dependent variable of patient satisfaction.

### 3.1 Socio‐Demographic Profiles

The demographic profiles of the 311 hospital visitors are summarized in Table 1. The majority of patients were women, comprising 70.1% of the total, whereas men comprised the remaining 29.9%. Patient ages ranged from 18 to over 60 years, with the 40–49 age group representing the largest segment and the 18–20 age group representing the smallest. The details of the age distribution are listed in Table 1.

**TABLE 1:** Frequency and percentage distribution of the respondents by their socio‐demographic characteristics (n = 311).

SN	Characteristics	Frequency	Percentage
**1**	**Gender**		
	Male	93	29.9
	Female	218	70.1
**2**	**Age group in years**		
	18–20	13	4.2
	21–29	51	16.4
	30–39	59	19.0
	40–49	74	23.8
	50–59	44	14.1
	60 and above	70	22.5
**3**	**Education status**		
	Illiterate	80	25.7
	Informal	70	22.5
	Primary level	86	27.7
	Basic level	35	11.3
	Secondary level	26	8.4
	Higher level	14	4.5
**4**	**Average household income per month**	A	
	Less than 10,000	198	63.7
	10,000–20,000	68	21.9
	20,000–30,000	24	7.7
	More than 30,000	21	6.8
**5**	**Number of hospital visit in 1 year**		
	1 time	19	6.1
	2 times	60	19.3
	3 times	68	21.9
	4 or more than 4 times	164	52.7
**6**	**OPD category**		
	Medical department	199	64
	General surgical department	26	8.4
	Obstetrics and gynecology department	50	16.1
	Dermatology and other departments	36	11.6

Abbreviation: OPD, outpatient department.

The patients’ educational backgrounds varied: 27.7% had completed primary education, 22.5% had no formal education, and 4.5% had higher education. Most patients (63.7%) had a monthly household income of less than 10,000, whereas 6.8% had incomes exceeding 30,000. More than half (52.7%) visited the hospital four or more times annually. Most patients (64%) were in the medical department, 8.4% in general surgery, 16.1% in obstetrics and gynecology, and 11.6% in dermatology and other departments (Table 1).

### 3.2 The Gap‐Model Analysis of Expectation and Perception

A gap model analysis was employed to evaluate the disparity between expectations and perceptions. This examination incorporated a component that displayed an attribute's mean scores for perception, expectation, and the resulting difference. By assessing these metrics, the study team can identify the aspects of SERVQUAL that need improvement to meet patient expectations.

Table 2 presents the outcomes of a gap model investigation that examined patient expectations and perceptions of hospital services. Participants were asked to assess their anticipation and observations of various elements of the hospital's service offerings. Average scores for perception, expectations, and their differences were calculated for each dimension. SERVQUAL is determined by subtracting expectations (*E*) from perceptions (*P*) (*P* − *E* = SQ). A negative SQ value signifies patient dissatisfaction, whereas a positive SQ value indicates patient satisfaction.

**TABLE 2:** Attribute with mean score of perception, expectation, and the gap.

Dimension and items	Mean perception score	Mean expectation score	Mean gap score
Tangible	4.265	4.277	−0.012
The hospital has modern‐looking equipment	4.34	4.27	+0.07
The hospital has visually appealing facilities	4.54	4.43	+0.11
Doctors and other employees have a professional appearance	4.17	4.21	−0.04
The hospital has appealing environment and materials associated with the service	4.01	4.20	−0.19
Reliability	3.99	4.24	−0.25
The hospital provides the services as promised	4.15	4.26	−0.11
It maintains error free records	3.86	4.15	−0.29
It performs the service right the first time	3.98	4.31	−0.33
The doctor gives enough time to consult and examine the patients	3.97	4.24	−0.27
Responsiveness	4.0625	4.34	−0.2775
Doctors and other employees offer prompt services to patients	4.03	4.27	−0.24
Doctors and other employees are willing to help patients	4.02	4.33	−0.31
Hospital employees always inform the patients exactly when services will be performed	4.12	4.35	−0.23
The doctors and nurses explain well about the nature of diseases and possible treatment options	4.08	4.41	−0.33
Assurance	4.176	4.354	−0.178
The hospitals are promptly able to handle patients’ problems	4.00	4.36	−0.36
Doctors and other employees are able to instill confidence in patients	4.41	4.50	−0.09
I feel safe in interactions with hospitals healthcare employees	4.25	4.32	−0.07
Assurance			
Doctors and other employees are courteous at all times	4.03	4.21	−0.18
Doctors and other employees have knowledge to answer questions from patients	4.19	4.38	−0.19
Empathy	4.118	4.332	−0.214
Doctors and other employees are polite and friendly dealing with patients	4.17	4.27	−0.10
Doctors and other employees have individualized attention to patients	4.03	4.28	−0.25
The hospital provides convenient consultation hours	4.09	4.36	−0.27
Doctors are dealing with patients with their ultimate care	4.16	4.39	−0.23

This study examined five dimensions: tangible, reliability, responsiveness, assurance, and empathy. In the tangible dimension, patients’ perceptions slightly exceeded their expectations, resulting in a minimal negative gap score (−0.012). This suggests that a hospital's physical aspects generally meet or surpass patient expectations.

The reliability dimension showed a more significant discrepancy, with a negative gap score (−0.25). Patients felt that the hospital's service delivery fell short of their expectations, particularly regarding accurate record‐keeping and first‐time SERVQUAL.

A negative gap score (−0.2775) in the responsiveness dimension indicates that patients find the hospital's services less timely and accommodating than anticipated. This is especially evident in the time doctors allocate for patient consultations and examinations, with a perceived gap of −0.27.

The assurance dimension also reveals a negative gap score (−0.178). Patients perceived the hospital's problem‐solving capabilities and the ability to instill confidence to be lower than expected. However, they generally feel secure in their interactions with the hospital staff.

Finally, the empathy dimension displayed a negative gap score (−0.214). The patients believed that the hospital's capacity to provide personalized attention and comprehend their specific needs was below their expectations. This is particularly noticeable in the convenience of consultation hours, where patients perceived a gap of −0.27.

### 3.3 Mean Score of Satisfaction

Table 3 shows the average ratings of patient contentment for various aspects of OPD visits to hospitals. The table includes statements assessing patient satisfaction and categorizes responses into “dissatisfied,” “neutral,” “satisfied,” and “very satisfied.” Additionally, it provides the number and percentage of patients who selected each response option, along with the overall mean score for each statement.

**TABLE 3:** Mean score of satisfaction.

Statement	Response level	Frequency	Percentage	Mean score
I am satisfied with the overall experiences in the hospital	Very dissatisfied	0	0	**4.15**
	Dissatisfied	6	1.9	
	Neutral	35	11.3	
	Satisfied	177	56.9	
	Very satisfied	93	29.9	
I am satisfied with the medical treatments which are successful	Very dissatisfied	0	0	**4.14**
	Dissatisfied	3	1	
	Neutral	31	10	
	Satisfied	198	63.7	
	Very satisfied	79	25.4	
I am satisfied as the medical services have fulfilled my requirement's	Very dissatisfied	0	0	**4.07**
	Dissatisfied	1	0.3	
	Neutral	53	17	
	Satisfied	178	57.2	
	Very satisfied	79	25.4	
I am satisfied with the overall performance of services provided by doctors, nurses and other employees	Very dissatisfied	0	0	**4.08**
	Dissatisfied	1	0.3	
	Neutral	46	14.8	
	Satisfied	191	61.4	
	Very satisfied	73	23.5	
I will recommend the hospital to other people	Very dissatisfied	0	0	**4.37**
	Dissatisfied	0	0	
	Neutral	25	8	
	Satisfied	145	46.6	
	Very satisfied	141	45.3	
**Satisfaction**				**4.162**

The survey indicated that 56.9% of the patients were satisfied with their overall hospital experience (mean score: 4.15). For medical treatments, 63.7% reported satisfaction (mean score: 4.14), and 57.2% were content with services that met their needs (mean score: 4.07). Healthcare staff performance was satisfactory in 61.4% of the patients (mean score: 4.08). Moreover, 46.6% of the patients recommended the hospital (mean score: 4.37). Overall, patient satisfaction averaged 4.162, indicating general contentment with hospital experiences, with no extreme dissatisfaction reported.

### 3.4 Correlations Between Each Variable

Table 4 illustrates the correlation between six variables, namely, satisfaction, tangible, reliability, responsiveness, assurance, and empathy, and how they are interrelated with each other. The correlation coefficient, ranging from −1 to 1, indicates the strength and direction of the relationship between two variables, with positive values suggesting that an increase in one variable tends to be associated with an increase in the other.

**TABLE 4:** Correlations among study variables.

		Satisfaction	Tangible	Reliability	Responsiveness	Assurance	Empathy
Satisfaction	Pearson correlation	1					
Tangible	Pearson Correlation	0.413^**^	1				
Reliability	Pearson correlation	0.447^**^	0.529^**^	1			
Responsiveness	Pearson correlation	0.472^**^	0.526^**^	0.747^**^	1		
Assurance	Pearson correlation	0.450^**^	0.520^**^	0.637^**^	0.758^**^	1	
Empathy	Pearson correlation	0.463^**^	0.509^**^	0.658^**^	0.801^**^	0.789^**^	1

** Correlation is significant at the 0.01 level (2‐tailed).

The results revealed robust positive correlations among all variables at a significance level of 0.01, demonstrating strong interconnections. The most pronounced correlation exists between empathy and responsiveness (*r* = 0.801**), with assurance and empathy (*r* = 0.789**), assurance and responsiveness (*r* = 0.758**), and reliability and responsiveness (*r* = 0.747**) following closely behind. These outcomes imply that patients who perceive high levels of empathy, responsiveness, assurance, and reliability tend to express satisfaction. Moreover, physical aspects, such as the appearance of facilities and equipment, showed a positive, albeit weaker, correlation with patient satisfaction.

### 3.5 The Coefficients Table for Multiple Linear Regression

Table 5 presents the multiple linear regression analysis examining the relationship between demographic variables and patient satisfaction. The unstandardized coefficients (*B*) show the change in satisfaction associated with a one‐unit increase in each variable while holding others constant. The standard error for each unstandardized coefficient is provided in the “std. error” column. Standardized coefficients (beta) indicate the change in satisfaction, with one standard deviation increase in each variable. The *t* values (*t*) test the null hypothesis that each unstandardized coefficient equals zero, with corresponding *p* values (Sig.) that were considered statistically significant. The constant coefficient represents the expected satisfaction level when all variables are zero.

**TABLE 5:** Multiple linear regression between demographic variables and satisfaction coefficients.

Model		Unstandardized coefficients		Standardized coefficients	*t*	Sig.
		*B*	Std. error	Beta		
1	(Constant)	20.890	0.999		20.917	0.000
	Gender	−0.199	0.292	−0.042	−0.680	0.497
	Age interval	0.092	0.113	0.064	0.811	0.418
	Education status	−0.349	0.127	−0.230	−2.747	0.006
	Average household income per month	0.257	0.169	0.107	1.521	0.129
	No of hospital visit in year	−0.104	0.128	−0.046	−0.814	0.416
	Disease category	0.433	0.119	0.222	3.644	0.000

The results revealed that education status and disease category were significantly associated with satisfaction (*p* < 0.01). Specifically, individuals with higher educational status and those in a higher OPD category tended to report higher levels of satisfaction. However, the other independent variables did not show significant associations with satisfaction, which may be due to insufficient statistical power or other unaccounted factors.

The regression model was statistically significant [*F*(6, 304) = 4.091, *p* = 0.001], indicating that the predictors are collectively related to satisfaction. The model explained a notable portion of the variance in satisfaction, with a regression sum of squares of 106.521. The mean square for the regression (17.754) also demonstrated that the predictors moderately explained the variance in satisfaction. The remaining variance was explained by the residuals, with a sum of squares of 1319.286.

Table 6 displays the ANOVA results from a multiple linear regression analysis of the relationship between the demographic variables and satisfaction (dependent variable). The model incorporated six predictors: OPD category, annual hospital visits, monthly household income, sex, age interval, and educational status. Overall, the ANOVA table indicated that the demographic variables significantly predicted satisfaction, explaining a moderate portion of the variance.

**TABLE 6:** ANOVA table for multiple linear regressions between demographic variables and satisfaction.

ANOVA^a^						
Model		Sum of squares	Df	Mean square	*F*	Sig.
1	Regression	106.521	6	17.754	4.091	0.001^b^
	Residual	1319.286	304	4.340		
	Total	1425.807	310			

^a^ Dependent variable: satisfaction.

^b^ Predictors: (constant), OPD category, no of hospital visit in a year, average household income per month, gender, age interval, education status.

### 3.6 Multiple Linear Regression Between SERVQUAL Dimensions and Satisfaction

Multiple linear regressions between SERVQUAL dimensions and satisfaction are presented, along with the ANOVA table. This section offers a deeper understanding of the relationship between SERVQUAL and patient satisfaction and identifies the factors that need improvement to enhance patient satisfaction.

Table 7 presents a statistical analysis of the correlation between patient satisfaction and five factors: tangibility, reliability, responsiveness, assurance, and empathy. The table provides the coefficients for each factor and the intercept. The intercept value of 9.27 represents the estimated average satisfaction when all factors are zero. The standardized coefficient (beta) illustrates each factor's influence on satisfaction, considering the effects of other factors. The findings reveal that tangible has a significant positive impact on satisfaction (beta = 0.173, *t* = 2.885, *p* = 0.004), suggesting that enhanced physical facilities, equipment, and staff appearance contribute to increased patient satisfaction. Reliability had a positive but statistically insignificant effect (beta = 0.129, *t* = 1.700, *p* = 0.090), indicating a potential but uncertain contribution to satisfaction. Responsiveness, assurance, and empathy did not demonstrate significant effects on satisfaction (*p* > 0.05), implying that they may not be crucial predictors in this particular context. In summary, among these dimensions, tangible factors emerged as the most significant predictors of patient satisfaction, although it is important to note that these results are context‐specific and may not be generalizable to other populations or situations.

**TABLE 7:** Multiple linear regression between service quality dimensions and satisfaction.

Coefficients^a^						
		Unstandardized coefficients		Standardized coefficients		
Model		*B*	Std. error	Beta	*t*	Sig.
1	(Constant)	9.270	1.184		7.830	0.000
	Tangible	0.228	0.079	0.173	2.885	0.004
	Reliability	0.126	0.074	0.129	1.700	0.090
	Responsiveness	0.108	0.090	0.116	1.204	0.229
	Assurance	0.082	0.077	0.091	1.065	0.288
	Empathy	0.105	0.077	0.126	1.365	0.173

^a^ Dependent variable: satisfaction.

An ANOVA derived from multiple linear regression analysis is presented in Table 8, illustrating the connection between patient satisfaction and five dimensions: tangibility, reliability, responsiveness, assurance, and empathy. The regression sum of squares (403.175) demonstrated that these five predictors substantially accounted for the variation in patient satisfaction. The model's statistical significance was confirmed by an *F*‐statistic of 24.049 and a *p* value of 0.000 (less than 0.05). After considering the predictors, the residual sum of squares (1022.632) represented unexplained variability in patient satisfaction. The mean square (3.353) indicates the average unexplained variance per degree of freedom (DOF). The total sum of squares (1425.807) encompassed the overall variability in patient satisfaction scores. These results indicate that these five dimensions have a significant impact on patient satisfaction. Table 9 reveals that the entire analysis reached an *R* ^2^ value of 0.283, with *F*(5, 403.175) = 24.049 and *p* ≤ 0.001. The dependent variable (satisfaction) is explained or affected by 28.3% of the variance by the independent variables.

**TABLE 8:** ANOVA table for multiple linear regressions between service quality dimensions and satisfaction.

ANOVA^a^						
Model		Sum of squares	Df	Mean square	*F*	Sig.
1	Regression	403.175	5	80.635	24.049	0.000^b^
	Residual	1022.632	305	3.353		
	Total	1425.807	310			

^a^ Dependent variable: satisfaction.

^b^ Predictors: (Constant), tangible, reliability, responsiveness, assurance, and empathy.

**TABLE 9:** Model summary.

Model	*R*	*R* ^2^	Adjusted *R* ^2^	Std. error of the estimate
1	0.532^a^	0.283	0.271	1.83109

^a^ Predictors: (Constant), empathy, tangible, reliability, assurance, responsiveness.

## 4 Discussion

This study examined the connection between patients’ anticipation and impression of healthcare services and their overall contentment with hospital stay. These findings suggest that patient satisfaction with their hospital experiences is strongly linked to their perceptions of healthcare services. Consistent with previous research, this study identified reliability, responsiveness, assurance, and empathy as crucial factors that influence patient satisfaction. Additionally, the investigation revealed that tangible aspects, such as modern and visually appealing facilities, equipment, and personnel, significantly contribute to patient contentment. The research outcomes indicated that the physical appearance of buildings, equipment, and staff plays a substantial role in shaping patients’ perceptions of healthcare services and their overall satisfaction with the hospital experience.

The analysis of patient expectations and perceptions in a hospital using the gap model provides crucial insights into patients’ perceptions of the hospital's services and how they compare them with their expectations. This study emphasizes the need for hospitals to address the gaps between perceptions and expectations, especially in the areas of reliability, responsiveness, assurance, and empathy. The current study's findings are in line with those of previous research that investigated the gap between patients’ perceptions and expectations of healthcare services. For instance, [11] identified similar dimensions of SERVQUAL and found that patients’ expectations were higher than their perceptions, resulting in a negative gap score.

Regarding the tangible aspect, this study's results align with earlier research highlighting the significance of contemporary and aesthetically pleasing healthcare facilities [12]. However, a negative gap score indicates that patient expectations in this area still exceed current standards, suggesting the potential for enhancement. The reliability dimension exhibited the largest negative gap score, revealing a considerable discrepancy between patient expectations and the hospital's perceived ability to fulfill them. This finding corroborates with previous studies that have identified reliability as a crucial factor influencing patient satisfaction [13].

The responsiveness dimension also exhibits a considerable negative gap score, suggesting that patients find the hospital's services less timely and supportive than anticipated. This aligns with earlier studies that highlight responsiveness as a crucial element in patient contentment [14]. Additionally, the assurance dimension shows a negative gap score, implying that patients perceive the hospital's capacity to address issues and build trust as falling short of their expectations. This finding is consistent with previous research that emphasized assurance as a vital component of patient satisfaction [11]. Finally, the empathy dimension also demonstrated a negative gap score, indicating that patients viewed the hospital's ability to offer personalized attention and comprehend their unique requirements as inferior to their expectations.

This study revealed a strong correlation between patients’ views on hospital services and their overall contentment with their hospital stay. This study identified key factors influencing patient satisfaction, including hospital responsiveness, assurance, empathy, and reliability. Furthermore, concrete elements of care, such as the hospital's physical environment and amenities, were found to significantly impact patient satisfaction. These outcomes align with the findings of the current investigation, which similarly recognized empathy, responsiveness, assurance, and reliability as crucial determinants of patient contentment. Additionally, the present study emphasized the role of tangible aspects of care in shaping patient satisfaction. The observation that these concrete elements of care substantially influence patient satisfaction is also in line with the earlier research conducted by [15].

Multiple linear regression analysis revealed that educational status and OPD category were significantly associated with satisfaction. These outcomes align with earlier studies that have demonstrated education status as an indicator of patient satisfaction [15] and disease category as a crucial element influencing patient satisfaction [16].

This study investigated the relationship between patient satisfaction and five dimensions (tangibility, reliability, responsiveness, assurance, and empathy) in a specific setting. The findings revealed that tangible aspects significantly and positively influenced satisfaction, whereas reliability had a positive but statistically insignificant effect. Responsiveness, assurance, and empathy did not demonstrate significant impacts on satisfaction, indicating that they may not be crucial determinants of satisfaction. This study underscores the significance of the physical appearance of facilities, equipment, and staff in shaping patient satisfaction. These outcomes align with those of earlier research that identified tangible factors as key predictors of patient satisfaction [11]. However, the absence of significant effects for reliability, responsiveness, and assurance in this study contradicts previous findings that have recognized these dimensions as important predictors of satisfaction [17].

Although this study found that reliability, responsiveness, and assurance did not significantly predict patient satisfaction, this result is at odds with earlier studies conducted in various healthcare settings [11, 13, 14]. One reason for this discrepancy could be the study environment, as the data were gathered from a single public hospital where patients might value tangible and visible aspects of care, such as facilities, equipment, and staff appearance, more than relational or process‐oriented dimensions. In public institutions with limited resources, patients may adjust their expectations regarding timeliness, accuracy, and assurance of services, which could reduce the impact of these factors on their satisfaction levels. In addition, cultural and contextual differences may influence how patients perceive SERVQUAL. In Nepal, improvements in infrastructure and tangible facilities may be more closely linked to satisfaction than interpersonal or procedural elements. This variation underscores the need to adapt SERVQUAL models to different healthcare contexts and suggests that strategies to enhance patient satisfaction in similar settings should equally emphasize both tangible and intangible aspects of service.

A hospital‐based cross‐sectional study was conducted at the Hetauda Provincial Hospital in Nepal, where the sample size was calculated using the satisfaction level of a previous study conducted in a similar setting in Nepal, in which a valid tool, the SERVQUAL tool, was used to mitigate bias and strengthen the validity and generalizability of the study findings. However, it did not represent all public hospitals in Nepal and was conducted using quantitative methods. Drawing from the study results, several suggestions can be offered to hospital administrators and policymakers in Nepal. First, there should be a stronger focus on enhancing the tangible elements of healthcare services, such as investing in modern infrastructure, aesthetically pleasing facilities, and up‐to‐date medical equipment. These initiatives would not only improve the physical setting of hospitals but also positively affect patients’ perceptions of the care they receive. The Government of Nepal has already embarked on hospital improvement and modernization projects in accordance with its National Health Policy, and extending these efforts to provincial and district hospitals could further boost patient satisfaction [1, 6]. Second, the reliability aspect can be enhanced by systematically training staff in accurate record‐keeping, error‐free documentation, and consistent service delivery. By strengthening hospital information systems and offering refresher training for healthcare providers, service gaps can be minimized, thereby improving overall reliability [15]. Lastly, incorporating regular monitoring and patient feedback systems would enable administrators to track progress, address shortcomings, and continuously align services with patient expectations, as recommended in global healthcare quality frameworks [4, 6].

This research has several significant strengths, such as employing the validated SERVQUAL model, utilizing a statistically sufficient sample size, and implementing systematic random sampling, all of which bolstered the reliability and representativeness of the results. The use of correlation and regression analyses adds further depth to this interpretation. Nonetheless, the study's focus on a single hospital in Bagmati Province limits its generalizability. Its cross‐sectional design hinders the ability to draw causal conclusions, and dependence on self‐reported quantitative data may have led to recall or social desirability bias. Additionally, other contextual elements, such as cultural perceptions and provider workload, were not evaluated.

## 5 Conclusion

In conclusion, the application of the gap model to evaluate patient expectations and perceptions in hospital settings is vital for pinpointing areas that need improvement to increase patient satisfaction. This study demonstrated that patients’ assessments of hospital services were significantly linked to their overall satisfaction, with reliability, responsiveness, assurance, empathy, and tangible care aspects emerging as critical predictors. These results corroborate those of previous studies, underscoring the need to address disparities between patient expectations and perceptions, particularly in these key areas. Enhancing these aspects can improve patient satisfaction and healthcare outcomes. Therefore, it is imperative for healthcare providers to emphasize patient‐centered care by continuously assessing and refining their services to fulfill patient expectations and deliver a satisfactory experience. These insights contribute to a deeper understanding of the factors influencing patient satisfaction in healthcare contexts and offer valuable guidance for improving SERVQUAL and patient outcomes.

## Author Contributions

**Vivek Uprety**: conceptualization, methodology, investigation, data collection, software, formal analysis, writing original manuscript draft. **Sunil Neupane, Hemanta Neupane, Manoj Sigdel,** and **Mallika Shrestha**: investigation, validation, data curation, manuscript review, and editing. **Binita Kumari Paudel**: conceptualization, supervision, project administration, manuscript review, and editing. **Sagun Kharel** and **Bipindra Pandey**: software, formal analysis, data curation, investigation, resources, validation, manuscript review, and editing.

## Funding

The authors have nothing to report.

## Ethics Statement

Ethical approval was received from the Institutional Review Committee of the Purbanchal University School of Health Sciences, Purbanchal University, Morang, Nepal (Ref. no. 004‐079/80), before the commencement of the study.

## Consent

The authors have nothing to report.

## Conflicts of Interest

The authors declare no conflicts of interest.

## Data Availability

All relevant data are in the article, and any query regarding the findings of this study can be obtained from the corresponding author upon request.
